# Benzolactam-related compounds promote apoptosis of HIV-infected human cells via protein kinase C–induced HIV latency reversal

**DOI:** 10.1074/jbc.RA118.005798

**Published:** 2018-11-09

**Authors:** Kouki Matsuda, Takuya Kobayakawa, Kiyoto Tsuchiya, Shin-ichiro Hattori, Wataru Nomura, Hiroyuki Gatanaga, Kazuhisa Yoshimura, Shinichi Oka, Yasuyuki Endo, Hirokazu Tamamura, Hiroaki Mitsuya, Kenji Maeda

**Affiliations:** From the ‡ National Center for Global Health and Medicine Research Institute, Tokyo 162-8655, Japan,; the § Institute of Biomaterials and Bioengineering, Tokyo Medical and Dental University, Tokyo, 101-0062, Japan,; the ¶ AIDS Clinical Center, National Center for Global Health and Medicine, Tokyo 162-8655, Japan,; the ‖ AIDS Research Centre, National Institute of Infectious Diseases, Tokyo 162-8640, Japan,; the ** Center for AIDS Research, Kumamoto University, Kumamoto 860-0811, Japan,; the ‡‡ Faculty of Pharmaceutical Sciences, Tohoku Medical and Pharmaceutical University, Sendai 981-8558, Japan, and; the §§ Experimental Retrovirology Section, HIV and AIDS Malignancy Branch, NCI, National Institutes of Health, Bethesda, Maryland 20892-1868

**Keywords:** apoptosis, retrovirus, human immunodeficiency virus (HIV), antiviral agent, protein kinase C (PKC), benzolactam, HIV cure, latency-reversing agents, latent infection, PKC activator

## Abstract

Latency-reversing agents (LRAs) are considered a potential strategy for curing cells of HIV-1 infection. Certain protein kinase C (PKC) activators have been previously reported to be LRAs because they can reverse HIV latency. In the present study, we examined the activities of a panel of benzolactam derivatives against cells latently infected with HIV. Using determination of p24 antigen in cell supernatants or altered intracellular GFP expression to measure HIV reactivation from latently infected cells along with a cytotoxicity assay, we found that some of the compounds exhibited latency-reversing activity, which was followed by enhanced release of HIV particles from the cells. One derivative, BL-V8-310, displayed activity in ACH-2 and J-Lat cells latently infected with HIV at a concentration of 10 nm or higher, which was superior to the activity of another highly active PKC activator, prostratin. These results were confirmed with peripheral blood cells from HIV-infected patients. We also found that these drugs up-regulate the expression of caspase 3 and enhance apoptosis specifically in latently HIV-infected cells. Moreover, combining BL-V8-310 with a bromodomain-containing 4 (BRD4) inhibitor, JQ1, not only enhanced HIV latency-reversing activity, but also reduced the effect on cytotoxic cytokine secretion from CD4^+^ T-cells induced by BL-V8-310 alone. Our results suggest that BL-V8-310 and its related benzolactam derivatives are potential LRA lead compounds that are effective in reversing HIV latency and reducing viral reservoirs in HIV-positive individuals with few adverse effects.

## Introduction

Prolonged anti-retroviral therapy (ART)^4^ suppresses human immunodeficiency virus (HIV) replication; however, even lifelong ART cannot completely eradicate HIV from the body of patients because of persistent latent cell reservoirs (1–5). Hence, latency-reversing agents (LRAs) are considered a potential tool to cure HIV, and a number of groups have reported molecules that reactivate cells latently infected with HIV. The approach to eliminate HIV reservoir cells using LRAs is called “shock and kill” (6–8). However, developing safe drugs with no/minimal effect on HIV-uninfected cells appears to be challenging. Moreover, recent clinical studies have demonstrated that certain LRAs activate HIV gene expression *in vivo*, but with limited or no clearance of reactivated cells (9–11). This is presumably because multiple mechanisms are involved in the maintenance of HIV latency (12); in fact, a recent study showed that T-cell activation does not induce all functionally latent proviruses and that a significant proportion of these noninduced proviruses are replication-competent (13).

Many small molecule agents that are currently being developed as LRAs include protein kinase C (PKC) activators (*e.g.* PEP005 (ingenol-3-angelate), prostratin, and bryostatin-1), HDAC inhibitors (*e.g.* SAHA/vorinostat), or BRD4 inhibitors (*e.g.* JQ1) (14–17). PKC is a family of at least 10 related serine/threonine kinases with different tissue distributions and cofactor requirements. It is well-established that these PKC isozymes play a critical role in the regulation of cell growth, differentiation, and apoptosis (18, 19). PKC activators induce the activation of transcription factors such as NF-κB, which binds to HIV–long-terminal repeat and thus activates HIV mRNA transcription (20). In addition, it is known that the potency of PKC activators as LRAs is strongly enhanced in combination with an LRA in another class. Several groups have previously reported that combined treatment is important for LRAs to obtain maximum reactivation (16, 17, 21). Among these combinations, JQ1 plus a PKC activator is considered to be the most effective combination (21). However, as candidates for LRAs, there are still serious concerns with PKC activators because PKC signaling has broad effects on cell metabolism, and thus, agents that target PKC signaling might be associated with multiple side effects. Hence, developing less toxic PKC activators that act as LRAs is an urgent matter.

Previously, Endo *et al.* (23–25) reported the synthesis and functional analyses of a panel of benzolactam derivatives (26) that have activity as PKC activators. Other groups also developed and reported other benzolactam derivatives (27, 28). Endo *et al.* (29) also showed that some of those drugs inhibited cell killing by HIV; however, the detailed mechanism associated with these molecules remains unknown.

In this study, we focused on the activity of these derivatives as LRAs via activation of PKC. We found that one benzolactam derivative, BL-V8-310, showed potent activity in reversing HIV latency without any cytotoxic events in cell lines and primary cells *in vitro*, especially in combination with a BRD4 inhibitor, JQ1.

## Results

### LRAs reactivate HIV latently-infected cell lines

**Figure 1.**
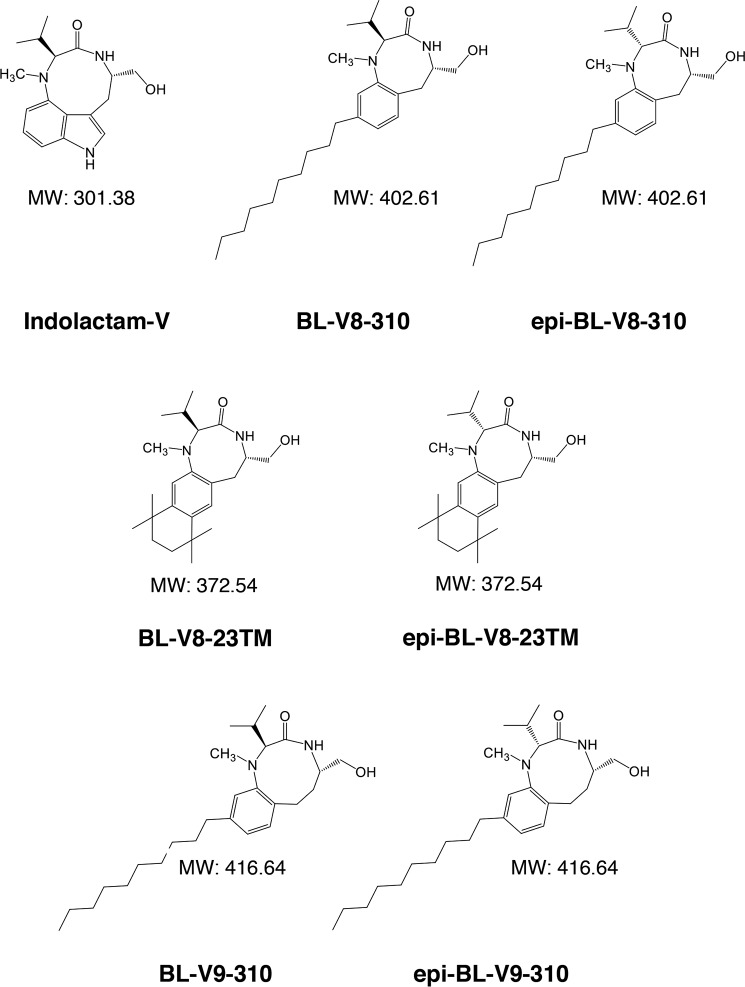
**Structure of benzolactam derivatives.**

**Figure 2.**
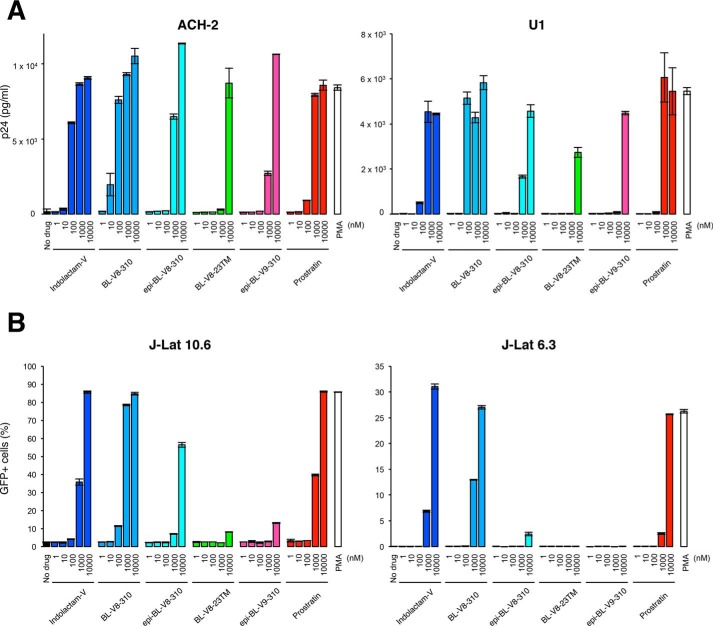
***In vitro* reversal of HIV latently-infected cells with benzolactam derivatives.** *A,* ACH-2 and U1 cells were exposed to a benzolactam derivative, and production of p24 in the supernatant was measured after a 48-h incubation. *B,* J-Lat 10.6 cells and J-Lat 6.3 cells (latently HIV infected cell lines) were exposed to a benzolactam derivative, and the change in the amount of GFP-positive cells was analyzed after 24 h by flow cytometry. Data are shown as means ± S.D. of three independent experiments.

In this study, we examined a panel of benzolactam derivatives (Fig. 1). Some of them reportedly have activity as PKC activators as candidates for novel LRAs. J-Lat cells and ACH-2 and U1 cell lines, which are latently infected with HIV, were treated with these benzolactam-related compounds. As shown in Fig. 2, five benzolactam compounds (Indolactam-V, BL-V8-310, epi-BL-V8-310, BL-V8–23TM, and epi-BL-V9–310) induced virus production in ACH-2 cells and U1 cells (Fig. 2*A*), whereas epi-BL-V8–23TM and BL-V9–310, optical isomers to BL-V8–23TM and epi-BL-V9–310, respectively (Fig. 1), failed to show activity. Reversal of latency in J-Lat 10.6 and J-Lat 6.3 cells was also examined by the increase of GFP-positive cells (Fig. 2*B*). Among these derivatives, BL-V8-310 (Fig. 1) was the most potent. BL-V8-310 induced HIV expression at 10 nm and higher in ACH-2 cells (Fig. 2*A*). Prostratin (16) and indolactam-V are PKC activators, which also induced the HIV-latency reversal, and BL-V8-310 was found to be similarly or more potent than these LRAs when examined with J-Lat (10.6 and 6.3), ACH-2, and U1 cells (Fig. 2). When EC_50_ values were calculated using the maximum reaction level (determined with excess of PMA) as 100% in ACH-2 cells, the EC_50_ value of BL-V8-310 was found to be 0.025 μm, which was lower than that of indolactam-V (0.047 μm) and prostratin (0.294 μm) (Table 1). Cytotoxicity of tested drugs was also examined using parental cell lines of ACH-2 cells and U1 cells (A3.01 and U937 cells, respectively), wherein BL-V8-310 was found to have only moderate cytotoxicity (*CC*_50_, 21.1 and 61.2 μm, respectively) (Table 1). BL-V8-310 also did not have toxicity in primary cells (*CC*_50_, 39.9 μm) (Table 1).

**Table 1:** **HIV latency reversal by benzolactam related compounds**

Drug	EC_50_*^a^*		CC_50_*^b^*			Apoptosis*^e^*	
	ACH-2	U1	A3.01*^c^*	U937*^c^*	PBMC*^d^*	ACH-2	U1
	μ*m*		μ*m*			%	
Indolactam-V	0.047 ± 0.001	0.36 ± 0.06	>100.0	>100.0	>100.0	47.1 ± 2.8	18.0 ± 0.4
BL-V8-310	0.025 ± 0.005	0.034 ± 0.002	21.1 ± 1.1	61.2 ± 2.2	39.9 ± 3.6	59.7 ± 2.3	30.4 ± 2.8
epi-BL-V8-310	0.43 ± 0.02	2.33 ± 0.11	17.8 ± 0.6	39.2 ± 0.3	18.9 ± 4.2	20.4 ± 3.0	6.1 ± 0.3
BL-V8-23TM	2.91 ± 0.35	9.89 ± 1.92	19.5 ± 2.8	22.9 ± 1.2	65.8 ± 0.5	ND	ND
epi-BL-V9-310	1.54 ± 0.05	4.00 ± 0.08	>100.0	>100.0	>100.0	ND	ND

Prostratin	0.294 ± 0.005	0.28 ± 0.06	>100.0	>100.0	>100.0	37.7 ± 4.2	14.1 ± 1.9

*^a^* The magnitude of reactivation induced by 10 nm PMA was defined as 100% reactivation, and concentrations of compounds giving 50% reactivation (viral production) were defined as EC_50_ values.

*^b^* Cell viabilities were determined by 3-(4,5-dimethylthiazol-2-yl)-2,5-diphenyltetrazolium assay at day 2. *CC*_50_, concentration of compound required to reduce the viability of parental cells by 50%.

*^c^* A3.01 and U937 cells are parental cell lines to ACH-2 and U1 cells, respectively.

*^d^* Cell viabilities of PBMC from healthy donor were determined by 3-(4,5-dimethylthiazol-2-yl)-2,5-diphenyltetrazolium assay at day 5.

*^e^* Apoptosis induction (with 1 μm of a drug) was detected by flow cytometry using PI/annexin-V staining. The average of two independent experiments is shown in Fig. 5*B*, *white bars*.

### HIV latency reversal is enhanced by combining BL-V8-310 with other LRAs

**Figure 3.**
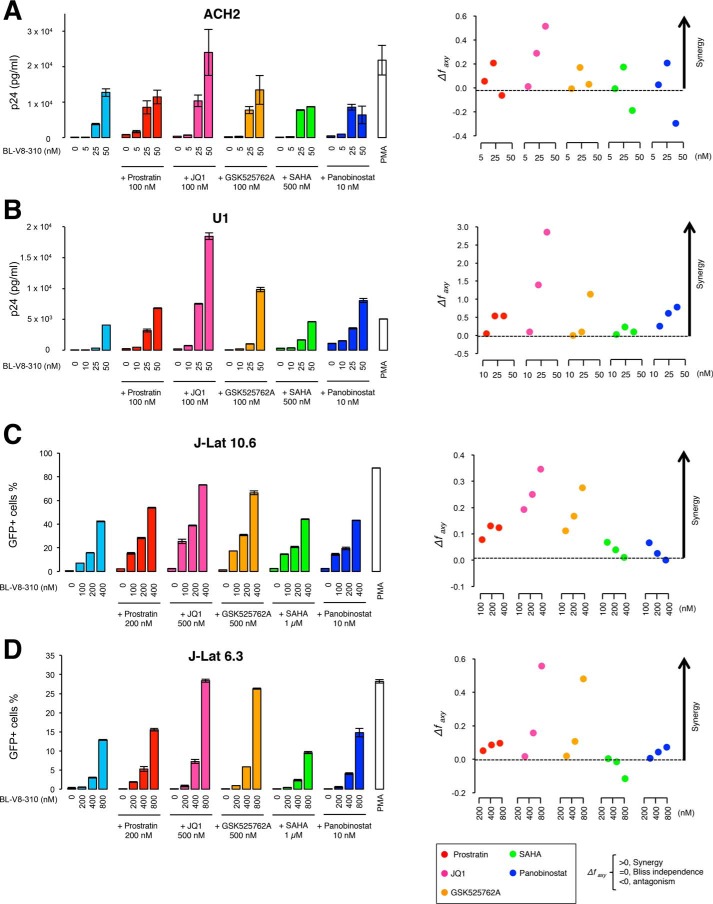
**Effect of combining BL-V8-310 with other LRAs on the reactivation of latent HIV.** *A,* ACH-2 cells, and *B,* U1 cells were treated with BL-V8-310 (5–50 nm) alone or in combination with prostratin (100 nm), JQ1 (100 nm), GSK525762A (100 nm), SAHA (500 nm), or panobinostat (100 nm) for 48 h, and supernatant p24 was measured. *C,* J-Lat 10.6 cells, and *D,* J-Lat 6.3 cells were treated with BL-V8-310 (100–400 nm) alone or in combination with prostratin (200 nm), JQ1 (500 nm), GSK525762A (500 nm), SAHA (1 μm), or panobinostat (10 nm) for 24 h, and the number of GFP-positive cells was measured. *A–D, right panels,* bliss independence model was utilized to define the synergism/antagonism of drug combinations. Synergism was defined as Δ*fa_xy_* > 0, and Δ*fa_xy_* < 0 indicated antagonism. Data are shown as means ± S.D. of three independent experiments.

Recent studies have shown that it is important to combine LRAs to obtain higher levels of HIV–RNA transcription (12, 17, 21, 30). For example, Jiang *et al.* (21), reported that PEP005 and JQ1 exhibit synergism in the reactivation of latent HIV (7.5-fold higher than PEP005 alone). Lu *et al.* (31) also reported that a PKC activator shows greater activity when combined with a BRD4 inhibitor, including JQ1. Thus, we examined the effect of combining BL-V8-310 with known LRAs on the reactivation of HIV in latent cells (Fig. 3). Prostratin (100 or 200 nm), JQ1 (100 or 500 nm), GSK525762A (BRD4 inhibitor) (100 or 500 nm), SAHA (500 nm or 1 μm), and panobinostat (HDAC inhibitor) (10 nm) were combined with various concentrations of BL-V8-310, and the increase of HIV production in ACH-2 and U1 cells (Fig. 3, *A* and *B*) or changes in the ratio of GFP-positive cells in J-Lat cells (Fig. 3, *C* and *D*) were determined. As for the drug concentrations that are used for the combination assay (Fig. 3), we determined a concentration of each drug that started to show LRA activity (>10% reactivation) in each cell line (data for ACH2 and J-Lat10.6 are shown in Fig. S1), and two drugs were combined with the concentrations. As shown in Fig. 3, *A–D*, treatment with BL-V8-310 alone (*light-blue bars*) increased supernatant p24 or GFP^+^ cells in concentration-dependent manner. In contrast, treatment with another LRA (prostratin, JQ1, GSK525762A, SAHA, and panobinostat at indicated concentrations) alone did not increase supernatant p24 or GFP^+^ cells. However, once they are combined with BL-V8-310, they (especially JQ1) enhanced the effect of BL-V8-310. For example, in Fig. 3*C*, single treatment of BL-V8-310 at 400 nm increased the GFP^+^ cell ratio to 42.2%, but when it was combined with 500 nm JQ1, the value increased to 73.1%. A similar result was observed when combined with GSK525762A in J-Lat 10.6 cells (Fig. 3*C*), but the addition of GSK525762A to BL-V8-310 did not affect the change in the supernatant p24 levels in ACH-2 cells (Fig. 3*A*). Overall, the enhancement of HIV reactivation was most obvious when BL-V8-310 was combined with JQ1 in all tested cells. Especially in U1 cells, 25 or 50 nm BL-V8-310 combined with JQ1 (100 nm) induced 22.9- and 4.5-fold enhanced reactivation, respectively, compared without JQ1 (Fig. 3*B*). The combination effects were analyzed using a Bliss independence model (17) (Fig. 3, *right panels*), and we found that the combination of BL-V8-310 and JQ1 (100 or 500 nm) had the most potent synergistic effect (Δ*f*_axy_ = 0.35–2.85; Δ*f*_axy_ > 0 defined as synergism) in all tested cells.

### BL-V8-310 reverses HIV latency in primary CD4^+^ T-cells from HIV-infected individuals

**Figure 4.**
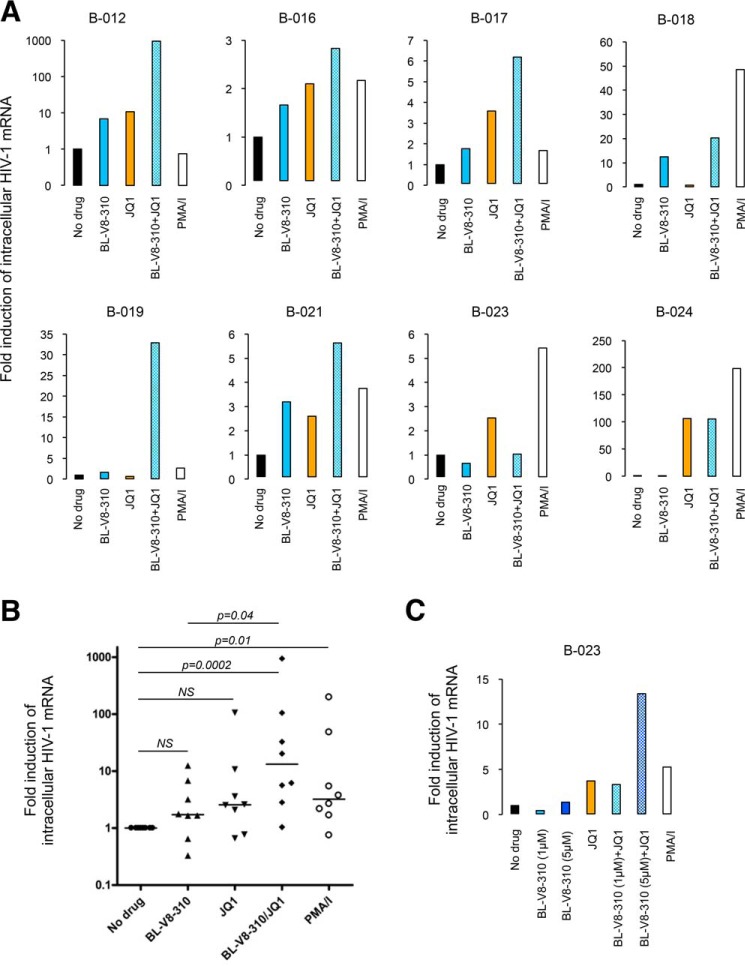
**BL-V8-310 reactivates HIV in CD4^+^ T-cells from HIV patients.** *A* and *B,* human CD4^+^ T-cells purified from eight HIV-infected patients treated with cART (Table 2) were treated with 1 μm BL-V8-310, 1 μm JQ1, combination of BL-V8-310 and JQ1, or 100 nm PMA plus 2 μm ionomycin for 24 h. *C,* CD4^+^ T-cells purified from patient 23 were treated with 1 or 5 μm BL-V8-310, 1 μm JQ1, combination of 1 or 5 μm of BL-V8-310 and JQ1 for 24 h. Intracellular HIV mRNA levels were detected by qRT-PCR and compared with those in untreated controls. Statistical significance was determined using Mann-Whitney *U* test, where *p* value <0.05 considered significant. Data are shown as medians of eight individuals.

We then examined the effect of BL-V8-310 using primary CD4^+^ T-cells from HIV-infected individuals treated with cART (Table 2). Primary cells from HIV-infected patients were treated with either 1 μm BL-V8-310 or 100 nm PMA plus 2 μm ionomycin for 24 h, and the level of HIV mRNA was measured. Single treatment of BL-V8-310 enhanced the transcription of HIV mRNA in CD4^+^ T-cells in six (out of eight) patients. JQ1 alone also enhanced the HIV mRNA level in six patients. In contrast, combination of BL-V8-310 and JQ1 elevated HIV mRNA in all patient samples (Fig. 4*A*). The data were summarized in Fig. 4*B*, and the increase with either BL-V8-310 or JQ1 was not significant compared with that of unstimulated cells, but the increase of mRNA with their combination (13.2-fold, compared with those without drug) was significant (*p* = 0.0002) (Fig. 4*B*). When the data (in Fig. 4*B*) were analyzed with the “Bliss independence” model, the calculated Δ*fa_xy_* value was 4.73, which is defined as a very strong synergism. Additionally, we examined higher concentrations of BL-V8-310 for patient B-023 (no response to 1 μm BL-V8-310 in Fig. 4*A*). When the cells were treated with 5 μm BL-V8-310, the transcription of HIV mRNA was increased by 1.4-fold. Combination with JQ1 also exhibited strong enhancement of HIV mRNA (by 13.4-fold) (Fig. 4*C*).

**Table 2:** **Clinical characteristics of patients employed in this study**

Patient	Male (M)/female (F)	Age	VL*^a^*	CD4 count*^a^*	cART*^b^*	Therapy	Plasma HIV RNA <20 copies/ml
		*years*	*copies/ml*	*cells/mm^3^*		*years*	*years*
B-012	F	44	<20	641	FTC/TAF/DRV/RTV	12	7
B-016	M	45	<20	1066	FTC/TAF/COBI/EVG	20	6
B-017	M	52	<20	1130	FTC/TAF/COBI/DRV	12	6
B-018	M	46	<20	440	ABC/3TC/ETR/RAL	22	7
B-019	M	51	<20	588	FTC/TAF/COBI/EVG	21	6
B-021	M	48	<20	915	FTC/TAF/COBI/EVG	13	5
B-023	M	51	<20	889	DRV/COBI/DTG	18	7
B-024	M	49	<20	465	ABC/3TC/DTG	19	6

*^a^* VL and CD4 count was done at the time of the study.

*^b^* The following abbreviations are used: 3TC, lamivudine; ABC, abacavir; COBI, cobicistat; DRV, darunavir; ETR, etravirine; EVG, elvitegravir; DTG, dolutegravir; FTC, embricitabine; RAL, raltegravir; TAF, tenofovir alafenamide fumarate.

### BL-V8-310 enhances apoptosis in HIV latent cells through caspase-3 signaling

**Figure 5.**
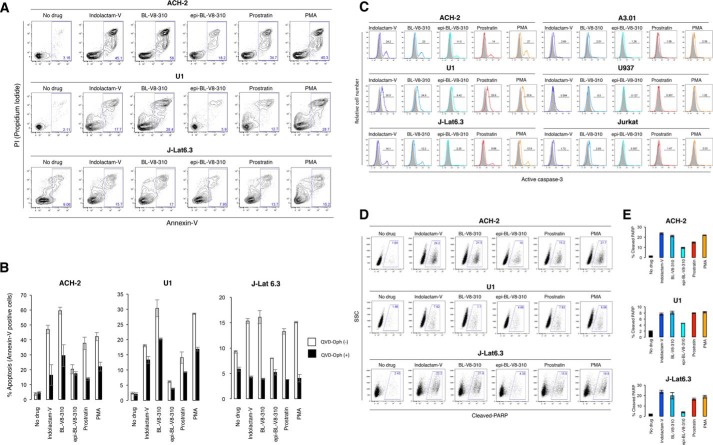
**Benzolactam derivatives induce apoptosis via caspase-3 activation in latently HIV-infected cells.** *A,* ACH-2, U1, and J-Lat 6.3 cells were exposed to 1 μm benzolactam compounds and cultured for 48 h. Cell apoptosis was analyzed by annexin-V/propidium iodide staining using flow cytometry. *B,* ACH-2, U1, and J-Lat 6.3 cells were exposed to 1 μm benzolactam compounds with 2-h pretreatment of a pan-caspase inhibitor (Q-VD-Oph, 20 μm) and cultured for 48 h. Cell apoptosis (with/without Q-VD-Oph) was analyzed, and proportions of apoptotic cells are shown. Data represent the mean ± S.D. *C,* ACH-2, U1, and J-Lat 6.3 cells and their parental cells A3.01, U937, and Jurkat cells were exposed to 1 μm benzolactams, 1 μm prostratin, and 10 nm PMA for 24 h, and caspase-3 activation was measured by flow cytometry. *D* and *E,* ACH-2, U1, and J-Lat 6.3 cells were exposed to 1 μm benzolactams, 1 μm prostratin, and 10 nm PMA for 24 h. Subsequently the amount (%) of cleaved-PARP in cells was measured by flow cytometry. Data are shown as means ± S.D. of three independent experiments.

*In vivo*, it is thought that reactivated HIV latent cells are eliminated by immune systems such as cytotoxic T-lymphocyte. However, cell death (by viral cytopathicity) or apoptosis in such reactivated cells is regarded as another important mechanism to decrease HIV latent reservoirs (32, 33). Thus, we examined the effect of LRAs, including BL-V8-310, on the induction of apoptosis in HIV latently-infected cells (Fig. 5). First, the ability of PKC activators to induce apoptosis was examined by propidium iodide (PI)/annexin-V staining. All five tested agents (indolactam-V, BL-V8-310, epi-BL-V8-310, prostratin, and PMA) increased the proportion of annexin-V–positive cells in ACH-2, U1, and J-Lat 6.3 cells, with BL-V8-310 having the most potent effect (58, 28.4, and 17% in ACH-2, U1, and J-Lat 6.3 cells, respectively) (Fig. 5*A*). We then examined how BL-V8-310 induced apoptosis. Cells were pre-treated with a caspase inhibitor (Q-VD-Oph) and then exposed to a tested PKC activator. As shown in Fig. 5*B*, pre-treatment with Q-VD-Oph strongly reduced the proportion of annexin-V–positive cells in the presence of PKC activators, including BL-V8-310 in all tested cells, suggesting that the induction of apoptotic signaling by PKC activators is likely through activation of the caspase pathway. The effect of the caspase inhibitor was smallest in U1 cells (28.4 to 20%, annexin-V–positive cells with BL-V8-310), compared with that in ACH-2 and J-Lat 6.3 cells (Fig. 5*B*), and thus it is thought that additional factor(s) might be involved in the apoptosis of certain cells such as U1 cells.

Subsequently, we examined the active caspase-3 expression in the presence of drugs. The treatment with 1 μm BL-V8-310, indolactam-V, and prostratin increased the expression of active caspase-3 in ACH-2 cells (23.0, 24.2, and 14.0%, respectively) (Fig. 5*C*). The effects of these drugs were also examined in A3.01 cells, a parental cell line of ACH-2 cells, and no increase in caspase-3 expression was found with BL-V8-310, indolactam-V, and prostratin. U1 cells and J-Lat 6.3 cells also responded to these drugs (Fig. 5*C*). Another benzolactam derivative, epi-BL-V8-310, also up-regulated caspase-3 expression, but the increase was smaller compared with that with BL-V8-310 (Fig. 5*C*). The U937 and Jurkat cell lines, parental cells of U1 and J-Lat 6.3 cells, respectively, were also used to confirm that these responses are specific in U1 and J-Lat 6.3 cells (HIV-latent cells), and results similar to those observed for ACH-2 cells were obtained (Fig. 5*C*).

We also determined whether BL-V8-310 enhances signaling downstream of the caspase pathway. It is known that caspase-3 activation leads to the cleavage of poly-ADP-ribose polymerase (PARP), which is a critical step for apoptotic cell death (34, 35). As shown in Fig. 5, *D* and *E*, all tested drugs induced the cleavage of PARP in ACH-2, U1, and J-Lat 6.3 cells.

Taken together, the induction of cell death by PKC activators, including BL-V8-310, is considered to be more specific for HIV-latent cells than for uninfected cells.

**Figure 6.**
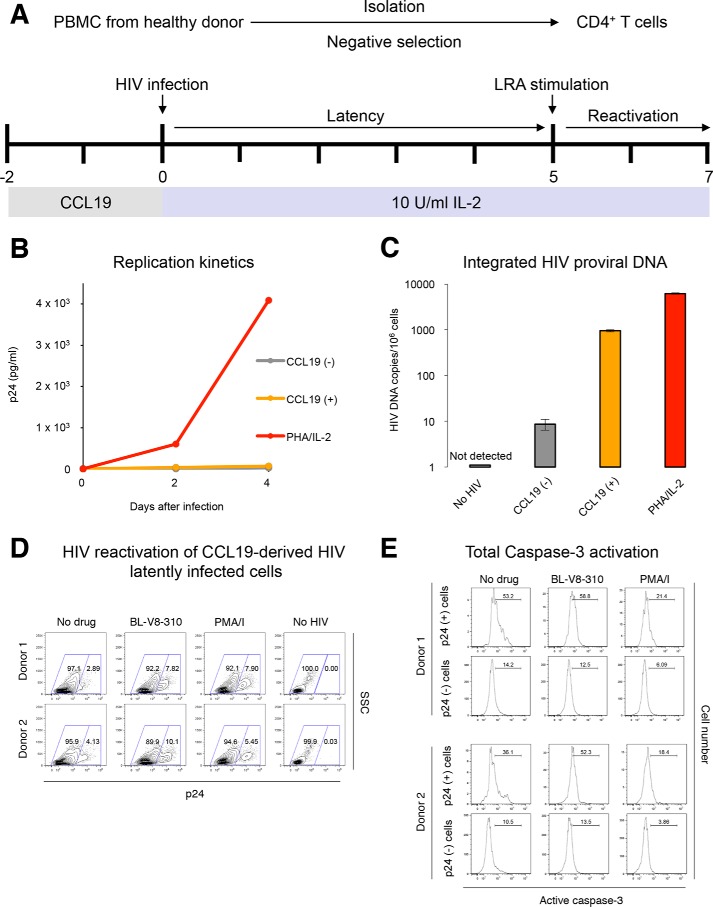
**Caspase-3 activation in a primary cell-derived, HIV latently-infected cell model.** *A,* protocol for the establishment of the CCL19-stimulated primary cell model of latent infection. *B,* HIV production from PBMCs (untreated, *gray*; CCL19-treated, *orange*; and phytohemagglutinin-stimulated, *red*) infected with HIV. *C,* amount of integrated HIV proviruses in the CCL19-treated/untreated PBMCs after infection. *D,* cell stimulation with BL-V8-310 or PMA/ionomycin. CCL19-treated primary cells were treated with BL-V8-310 (1 μm) or PMA (100 nm) plus ionomycin (*PMA/I*) (2 μm) for 48 h, and the change in the expression of intracellular p24 level was analyzed. Experiments were conducted using primary cells from two healthy donors (donors 1 and 2). *E,* up-regulation of the active caspase-3 with BL-V8-310 or PMA/ionomycin in HIV-infected CCL19-treated primary cells. Cells were stained with anti-p24 antibody, and changes in active caspase-3 expression in p24^+^ and p24^−^ populations were analyzed.

Finally, to examine the apoptotic effects of BL-V8-310 on human primary CD4^+^ T-cells latently infected with HIV, the CCL19-stimulated primary cell model of latent infection (36, 37) was employed. Peripheral blood mononuclear cells (PBMCs) from a healthy donor were treated with CCL19. Subsequently, CCL19-treated cells were infected with HIV and then cultured for 5 days (Fig. 6*A*). The CCL19-treated primary cells did not produce HIV when determined with a supernatant p24 value (Fig. 6*B*); however, we also found that the cells contain high levels of integrated HIV–DNA copies (954.6 copies/1 million cells). The amount of integrated HIV proviruses was lower than that in phytohemagglutinin-stimulated primary cells infected with HIV, but it was ∼2 log higher than that in unstimulated (CCL19(−)) PBMCs infected with HIV (Fig. 6*C*). Thus, it is considered that the CCL19-treated primary cells infected with HIV contain cells in which HIV provirus integrated without producing HIV; thus, the cell population is considered to be a “latent cell” population. The cells were then examined for the effects of stimulation with an LRA. Primary cells from two different donors were used for the experiments. It was found that PMA/I or BL-V8-310 treatment elevated the expression of intracellular p24 levels (after 48-h incubation). Treatment with BL-V8-310 increased the ratio of p24^+^ cells by 4.93% (donor 1) and 5.97% (donor 2), suggesting that the HIV-infected CCL19-treated cells contained 5–7% latent cell population (Fig. 6*D*). Using the cells, changes in the active caspase-3 induction were also examined. BL-V8-310 induced up-regulation of active caspase-3, and the increase of active caspase-3 was more obvious in p24^+^ cells (5.6 and 16.2%, respectively) than in p24^−^ cells (0 and 3.0%, respectively) (Fig. 6*E*). This result suggests that the treatment of BL-V8-310 increased the level of active caspase-3 specifically in HIV^+^ cells.

### Characterization of BL-V8-310 as a PKC activator and its effect on primary CD4^+^ T-cells

PKC plays a critical role in the regulation of cell growth, differentiation, and apoptosis (18, 19); thus, there are still serious concerns regarding the use of PKC activators for the treatment of latent HIV-infected reservoir cells. Hence, we characterized BL-V8-310 as a PKC activator and examined its detailed effect on primary CD4^+^ T-cells.

**Figure 7.**
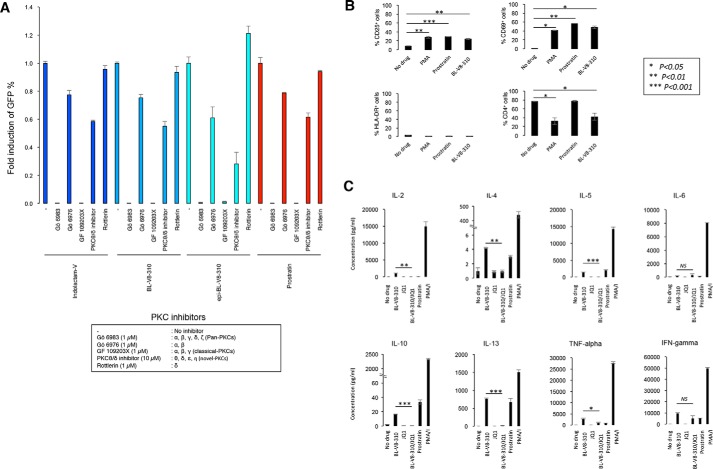
**PKC isozyme usage, T-cell activation profile, and the effect on primary CD4^+^ T-cells of PKC activators.** *A,* J-Lat 6.3 cells were treated with 10 μm benzolactam related compounds (indolactam-V, BL-V8-310, epi-BL-V8-310) for 24 h, with or without pretreatment of either Gö 6983 (1 μm), Gö 6976 (1 μm), GF 109203X (1 μm), PKCθ/δ inhibitor (10 μm), or Rottlerin (1 μm) for 3 h. Subsequently, GFP-positive J-Lat 6.3 cells were measured by flow cytometry. Data represent the mean ± S.D. *B,* primary CD4^+^ T-cells from healthy donors were treated with a drug for 24 h, and expressions of CD25, CD69, and HLA-DR (markers for broad T-cell activation) and CD4 were examined. *C,* analysis of the enhanced cytokine secretion from cells treated with LRAs. Primary CD4^+^ T-cells from healthy donors were treated with a drug or a combination (BL-V8-310 + JQ1), incubated for 24 h, and then concentrations of cytokines were measured using bead-based immunoassay. All assay was conducted in duplicate.

PKC consists of several isozymes, and to determine a major target isozyme of BL-V8-310, we conducted an HIV-latent cell reactivation assay with a panel of various PKC inhibitors. However, the tested benzolactam derivatives affected both a classical PKC inhibitor and a PKCθ/δ inhibitor, and the pattern of inhibitions indicated that BL-V8-310 targets both classical-PKCs (*e.g.* α, β, and γ) and novel PKCs (*e.g.* θ, δ, ϵ, and η) (Fig. 7*A*). It is noteworthy that many of the recently developed PKC activators, as LRA candidates, are known not to be tumor-promoting (22). We examined the activation profile of primary T-cells with BL-V8-310. Similarly to prostratin, BL-V8-310 elevated the ratio of CD25^+^ and CD69^+^ cells, which are markers for activated T-cells (Fig. 7*B*). The profile of the increased cell population was similar to that observed with prostratin, which is reportedly nontumor-promoting.

As shown in Table 1, BL-V8-310 had very minor cell toxicity *in vitro*; however, it is possible that the detailed activation profile of PKC activators on immune cells is invariably different. Thus, we examined the ability of BL-V8-310 and other LRAs to enhance the secretion of multiple cytokines when used alone or in combination. BL-V8-310 elevated supernatant cytokine concentrations of IL-2, IL-5, IL-6, IL-13, TNFα, and IFN-γ. However, when BL-V8-310 was combined with JQ1, the concentrations decreased compared with those with BL-V8-310 alone (Fig. 7*C*). To exclude the possibility that the reduction of cytokine secretion was due to the increased cell toxicity, we observed primary cells treated for 24 h with 1 μm BL-V8-310, 1 μm JQ1, and their combination (at 1 μm each) with PI staining, and we confirmed that the number of dead cells did not increase when treated with the combination (data not shown). The result suggests that the use of a PKC activator in combination with another LRA, such as JQ1, may not only enhance the HIV latency-reversing activity but also reduce the unfavorable effects of drugs on immune cells, which may help reduce the possibility of side effects occurring *in vivo*.

## Discussion

Recent studies have reported the development of a number of small molecule compounds that can reverse latent HIV infection (5, 14, 17, 21, 38), and PKC activators are potential candidate LRAs. In this study, we examined the detailed effect of a panel of benzolactam derivatives that were reported to be PKC activators (23–25). In a previous study, these benzolactam derivatives were suggested to have activity in suppressing acute HIV infection, and the associated mechanism was predicted to be the internalization of cell surface CD4 molecules (29). However, these were not further developed as conventional anti-HIV drugs. In the meantime, as presented in this study, these benzolactam derivatives reversed latently HIV-infected cells, and their potency was strongly enhanced by combined treatment with JQ1, which was similar to the previously reported studies using other PKC activators (17, 21). As shown in Fig. 4, treatment with BL-V8-310 alone failed to elevate HIV mRNA levels in two (of eight) PBMCs (patients 23 and 24), whereas the combination of BL-V8-310 and JQ1 effectively increased HIV mRNA level in seven (of eight) PBMCs (Fig. 4*A*). It is considered that the size of peripheral reservoir cells is different in each patient, even if all HIV patients are well controlled in viral load under cART (Table 2). Hence, the diversity of the reservoir size causes such difference of the response to LRAs *in vitro*. However, previous studies from other groups also demonstrated that some known small molecule LRAs did not show HIV-reversing activity when used alone in patient-derived PBMCs (17, 39). Thus, it is considered that the combined use (with a different class) of LRAs is important to obtain maximal activity.

PKC plays an important role in the regulation of cell growth, replication, and death (18, 19). BL-V8-310, which has only minor cell toxicity *in vitro*, also affected immune cell function (*e.g.* increase of cytokine secretion) (Fig. 7*C*). However, we also found that the combined treatment of BL-V8-310 with JQ1 reduced such an effect in primary cells (Fig. 7*C*), suggesting that the combined use of LRAs may contribute to reduce possible side effects *in vitro*.

Meanwhile, Lucera *et al.* (40) reported that vorinostat, a broad-spectrum HDAC inhibitor, increased the susceptibility of CD4^+^ T-cells to HIV and suggested that class I-specific HDAC inhibitors might reactivate latent cells without increasing the susceptibility of uninfected CD4^+^ T-cells to HIV. The result indicates the possibility that the further structural optimization of PKC activators, including BL-V8-310, and detailed characterization, such as the analysis of PKC isozyme usage, may enable the development of new PKC activators as more potent and less toxic LRA candidates.

Regarding the mechanism through which reactivated latent reservoir cells are eliminated, the production of HIV particles or HIV-related proteins in cells results in the capture of such reactivated cells by immune cells such as cytotoxic T-lymphocytes. However, apoptosis in reactivated HIV latent cells is also considered as another important mechanism through which these cells are eliminated (32). In fact, we previously showed that some LRAs such as PEP005 (PKC activator) induced apoptosis in HIV latently-infected cells (33). Thus, we believe that it is very important to investigate the effect of LRAs on “HIV latent cell–specific” cytotoxicity. In this regard, as shown in Figs. 5 and 6, we demonstrated that potent LRAs, including prostratin and BL-V8-310, induce caspase-3 activation followed by enhanced apoptosis in cells latently infected with HIV; furthermore, such marked induction was not observed in HIV-noninfected cells (*e.g.* Jurkat cells, parental cells of J-Lat cells) (Fig. 5). We also reported a mechanism of PKC-induced HIV reversal and cell apoptosis in HIV latent cells (33). PKC activation induces TNF receptor (TNF-R)–mediated NF-κB activation that causes high induction of viral transcription but simultaneously induces TNF-R–mediated caspase pathway activation (death signal) in HIV latent cells. The increase of viral protein production in cells by TNF-mediated NF-κB activation (referred as “survival signal”) also triggers apoptosis or cell cytopathicity induced by HIV-related proteins inside cells. Thus, it is considered that PKC activator-mediated apoptosis is caused by two different mechanisms: 1) direct caspase–pathway activation through TNF-R, and 2) indirectly mediated apoptosis/cell death by produced HIV proteins. The cytotoxicity of BL-V8-310 toward parental cells was moderate (Table 1), and thus it is possible that PKC activators, including BL-V8-310, can kill HIV-latent cells more specifically than HIV-noninfected cells. However, it is noteworthy that more detailed analyses for the mechanism of apoptosis and further experiments using primary CD4^+^ cell-derived models of latent HIV infection (Fig. 6) and animal models (*e.g.* HIV-infected humanized mice or SIV-infected macaques) will be necessary to determine whether LRAs can actually reduce the size of HIV reservoirs.

In summary, BL-V8-310 and its derivatives should be optimized to obtain potential candidate LRAs that are active against HIV reservoirs. Moreover, results in this study suggest the advantage of the combined use of LRAs, including PKC activators to reduce possible side effects *in vivo*.

## Experimental procedures

### Drugs and reagents

A panel of benzolactam derivatives, including BL-V8-310, was synthesized as described previously (23). Prostratin (PKC activator), SAHA/vorinostat, panobinostat (HDAC inhibitor), JQ1, and GSK525762A (BRD4 inhibitor) were purchased from Sigma, Santa Cruz Biotechnology (Dallas, TX), MedChem Express (Monmouth Junction, NJ), BioVision (Milpitas, CA), and ChemScene (Monmouth Junction, NJ), respectively. PMA was purchased from Wako Pure Chemical (Osaka, Japan). The pan-caspase inhibitor Q-VD-Oph was purchased from Tonbo Science (Kobe, Japan).

### HIV-latency reversal with LRAs

The reactivation of HIV from latently-infected cells was determined by quantifying p24 antigen in the supernatant (ACH-2 and U1 cells) or the changes in intracellular GFP expression (J-Lat cells). J-Lat cells (41, 42), ACH-2 cells, or U1 cells (5 × 10^5^ cells/ml) were placed in 96-well plates and incubated with different concentrations of drugs for 48 h to collect the supernatant. The increase in supernatant p24 antigen levels was measured using the Lumipulse G1200 (FUJIREBIO, Tokyo, Japan). J-Lat cells (5 × 10^5^ cells/ml) were placed in 48-well plates and incubated with different concentrations of a drug for 24 h. Then, GFP-positive cells were analyzed by flow cytometry.

### Cytotoxicity assays

To determine the cytotoxicity induced by LRAs, cells (5 × 10^5^ cells/ml) were cultured in the presence or absence of an LRA. After 48 h, cytotoxicity assays (WST-8 assay) were performed using a Cell Counting Kit-8 (Dojindo, Kumamoto, Japan) according to the manufacturer's instructions. The numbers of living cells after drug treatment were measured and compared with those in untreated cells and are shown as the % relative to the control.

### Primary CD4^+^ T-cell isolation from HIV patient samples and ex vivo reactivation

The isolation of primary cells from HIV^+^ patients and the *ex vivo* reactivation experiments were conducted as described previously (29). In brief, peripheral blood samples were collected from HIV-infected patients receiving cART for at least 5 years (Table 2). All subjects maintained a lower viral load (<20 copies/ml; except for occasional “blips”) during therapy. CD4^+^ T-cell counts in peripheral blood samples ranged from 465 to 1130 cells/mm^3^ (average, 767 cells/mm^3^), and plasma viral loads were <20 copies/ml as measured by qPCR (Roche Applied Science, COBAS AmpliPrep/COBAS TaqMan HIV-1 Test version 2.0) at the time of study enrollment. Written informed consent was obtained from all subjects. The Ethics Committee at the National Center for Global Health and Medicine approved this study (NCGM-G-002259-00), and each patient provided written informed consent. This study abided by the Declaration of Helsinki principles. Whole PBMCs were separated by density-gradient centrifugation with Ficoll-Paque^TM^ (GE Healthcare, Munich, Germany), and CD4^+^ T-cells were purified using the MojoSort^TM^ human CD4 T-cell isolation kit (BioLegend, San Diego) according to the manufacturer's instructions. Purified CD4^+^ T-cells were plated at a density of 2.5 × 10^6^ cells/ml and treated with 100 nm PMA plus 2 μm ionomycin, 1 μm BL-V8-310, 1 μm JQ1, or a combination for 24 h, and the cells were collected for RNA purification. Total RNA was extracted using an RNeasy mini kit (Qiagen, Hilden, Germany), following the manufacturer's protocols. cDNA was synthesized using PrimeScript RT Master Mix (Takara-bio, Shiga, Japan), and quantitative real-time PCR analyses for intracellular HIV RNA was carried out with PowerUp^TM^ SYBR Green Master Mix (Applied Biosystems, Foster City, CA). The oligonucleotide primers used were as follows: 5′-TGTGTGCCCGTCTGTTGTGT-3′ (forward), and 5′-GAGTCCTGCGTCGAGAGAGC-3′ (reverse) for HIV–RNA detection. HIV–RNA copy numbers were normalized to RNA input (21). The number of HIV–RNA copies was calculated using a standard curve obtained from serially-diluted HIV plasmids, and normalized values (HIV-RNA copies/input RNA (ng)) with each drug were compared with those without drug; the relative increase in HIV-1 RNA levels in the presence of a drug was then determined.

### Flow cytometric analysis

The determination of intracellular HIV p24, the active form of caspase-3, cleavage of PARP, and annexin-V/PI staining by flow cytometry were performed as described previously (43, 44). In brief, ACH-2, U1, or primary cells (2.5 × 10^5^ cells/ml) were fixed with 1% paraformaldehyde/PBS for 20 min and permeabilized with Flow Cytometry Perm Buffer (TONBO Biosciences, San Diego). After a 5-min incubation at room temperature, cells were stained with anti-HIV-1 p24 (24-4)-FITC monoclonal antibody (mAb) (Santa Cruz Biotechnology, Dallas, TX), Alexafluor 647-conjugated anti-active caspase-3 mAb (C92-605) (Pharmingen), FITC mouse anti-cleaved PARP mAb (Asp-214) (Pharmingen), or Alexafluor 647 mouse anti-cleaved PARP mAb (Asp-214) (Pharmingen) for 30 min on ice. For PI/annexin-V staining, cells were washed twice with PBS and resuspended in annexin-V binding buffer (BioLegend) at a concentration of 1 × 10^7^ cells/ml. Then, cells were stained with FITC (or APC) annexin-V (BioLegend) and propidium iodide solution (BioLegend) for 15 min at room temperature. For CD3, CD4, CD25, CD69, and HLA-DR cell-surface marker staining, cells were stained with Pacific Blue anti-human CD3 mAb (UCHT1), APC anti-human CD4 mAb (RPA-T4), PE anti-human CD25 mAb (BC96), PE anti-human CD69 mAb (FN50), and PE anti-human HLA-DR mAb (Tu36) (BioLegend) for 30 min on ice. Cells were then analyzed using a FACSVerse flow cytometer (BD Biosciences). The corrected data were analyzed with FlowJo software (Tree Star, San Carlos, CA).

### Primary cell model of HIV latency

The establishment of a primary cell model of HIV latency was performed as described previously (36, 37) with minor modifications. CD4^+^ T-cells were purified from fresh PBMCs obtained from healthy donors. Cells were maintained in medium supplemented with 100 nm CCL19 (MIP3-β) for 2 days. Then, cells were washed and infected with HIV-1_NL4-3_ (250 ng/ml p24) for 3 h at 37 °C. After infection, cells were washed three times and maintained in complete medium with 10 units/ml of IL-2 for 5 days. At 5 days post-infection, cells were collected for DNA purification, washed, and plated in a 96-well plate at a density of 2 × 10^6^ cells/ml. PMA (100 nm) and 2 μm ionomycin or 1 μm BL-V8-310 was added to each well. Unstimulated cells were used as a control. At 2 days post-stimulation, intracellular HIV p24 and active caspase-3 expression levels were analyzed by flow cytometry. Total cellular DNA in the CCL19-stimulated primary cells was extracted using a QIAmp DNA blood mini kit (Qiagen, Hilden, Germany), following the manufacturer's protocols. Quantitative real-time PCR analyses for the intracellular HIV-1 proviral DNA was carried out with Premix Ex Taq^TM^ (Probe qPCR) Rox plus (Takara-bio, Shiga, Japan). The oligonucleotide primers were used as follows: 5′-TGTGTGCCCGTCTGTTGTGT-3′ (forward), 5′-GAGTCCTGCGTCGAGAGAGC-3′ (reverse), and 6-carboxyfluorescein-CAGTGGCGCCCGAACAGGGA-BHQ-1 (probe) for HIV-1 proviral DNA detection. Subsequently, HIV proviral DNA copy was calculated with a standard curve generated with a serially diluted HIV-1_pNL4-3_ plasmid.

### Cytokine analysis

Purified CD4^+^ T-cells from healthy donors were treated with 100 nm PMA plus 2 μm ionomycin, 1 μm prostratin, 1 μm BL-V8-310, 1 μm JQ1, or a combination of BL-V8-310 and JQ1 for 24 h, and supernatants were harvested. Human IL-2, IL-4, IL-5, IL-6, IL-10, IL-13, TNFα, and IFN-γ in the supernatant were analyzed using flow cytometry bead-based immunoassay (LEGENDplex^TM^ Human Th1/Th2 Panel, BioLegend) according to the manufacturer's instructions.

### Statistical analyses

Differences between groups were analyzed for statistical significance using the Mann-Whitney *U* test. *p* values < 0.05 were considered statistically significantly different. Analysis was performed using GraphPad Prism software version 4 (La Jolla, CA). The Bliss independence model was utilized to determine synergism/antagonism of drug combinations (17). This model is defined by the equation *fa_xy, P_* = *fa_x_* + *fa_y_* − (*fa_x_*)(*fa_y_*), where *fa_xy, P_* is the predicted fraction affected by a combination of drug *x* and drug *y*, given the experimentally observed fraction affected for drug *x* (*fa_x_*) and drug *y* (*fa_y_*) individually. The experimentally observed fraction affected by a combination of drug *x* and drug *y* (*fa_xy, O_*) can be compared with the predicted fraction affected, which is computed using the Bliss model (*fa_xy, P_*) as follows: Δ*fa_xy_* = *fa_xy, O_* − *fa_xy, P_*. If Δ*fa_xy_* > 0 with statistical significance, then the combined effect of the two drugs exceeds that predicted by the Bliss model, and the drug combination displays synergy. If Δ*fa_xy_* = 0, then the drug combination follows the Bliss model for independent action. If Δ*fa_xy_* < 0 with statistical significance, then the combined effect of the two drugs is less than that predicted by the Bliss model, and the drug combination displays antagonism. In our analysis, the fraction affected was calculated as follows for the percentage of GFP-positive cells: *fa_x_* = (% GFP-positive cells after treatment with drug *x* − % GFP-positive cells in the absence of drug)/(% GFP-positive cells after treatment with PMA − % GFP-positive cells in the absence of drug).

## Author contributions

K. Matsuda, K. T., Y. E., and K. Maeda conceptualization; K. Matsuda, T. K., K. T., and K. Maeda resources; K. Matsuda, T. K., K. T., S.-i. H. H., W. N., and K. Maeda data curation; K. Matsuda and K. Maeda writing-original draft; T. K., K. T., S.-i. H. H., W. N., H. G., K. Y., S. O., H. T., and K. Maeda formal analysis; K. T., H. T., and K. Maeda funding acquisition; K. T. and K. Maeda validation; K. T., Y. E., H. T., and K. Maeda writing-review and editing; W. N., H. G., K. Y., S. O., Y. E., H. T., H. M., and K. Maeda supervision; K. Maeda methodology.

## Supplementary Material

Supporting Information

## Footnotes

4 The abbreviations used are:

ART: anti-retroviral therapy
cART: combined ART
LRA: latency-reversing agent
PKC: protein kinase C
PMA: phorbol 12-myristate 13-acetate
PARP: poly-ADP-ribose polymerase
PBMC: peripheral blood mononuclear cell
TNF: tumor necrosis factor
TNF-R: TNF receptor
PI: propidium iodide
HDAC: histone deacetylase
SAHA: suberoylanilide hydroxamic acid (or Vorinostat)
PE: phycoerythrin.
