# Supplementary material for: Benzolactam-related compounds promote apoptosis of HIV-infected human cells via protein kinase C–induced HIV latency reversal
Source: J Biol Chem. 2018 Nov 9;294(1):116–29. doi: 10.1074/jbc.RA118.005798 (PMC6322896; doi:10.1074/jbc.RA118.005798)
Supplement: Supporting Information [file supp_294_1_116__index.html]

Benzolactam-related compounds promote apoptosis of HIV-infected human cells via protein kinase C–induced HIV latency reversal — Benzolactam PKC Activators as Novel Latency-Reversing Agents — Benzolactam-related compounds promote apoptosis of HIV-infected human cells via protein kinase C–induced HIV latency reversal — Benzolactam PKC activators as novel latency-reversing agents — Supporting Information 

# Benzolactam-related compounds promote apoptosis of HIV-infected human cells via protein kinase C–induced HIV latency reversal

## Supporting Information

- Supporting Information (to be published online) Figure S1 - Figure S1
