# Supplementary material for: Benzolactam-related compounds promote apoptosis of HIV-infected human cells via protein kinase C–induced HIV latency reversal
Source: J Biol Chem. 2018 Nov 9;294(1):116–29. doi: 10.1074/jbc.RA118.005798 (PMC6322896; doi:10.1074/jbc.RA118.005798)
Supplement: Supporting Information [file supp_RA118.005798_140523_2_supp_231613_phmg43.pdf]

Supporting Information
Figure S1

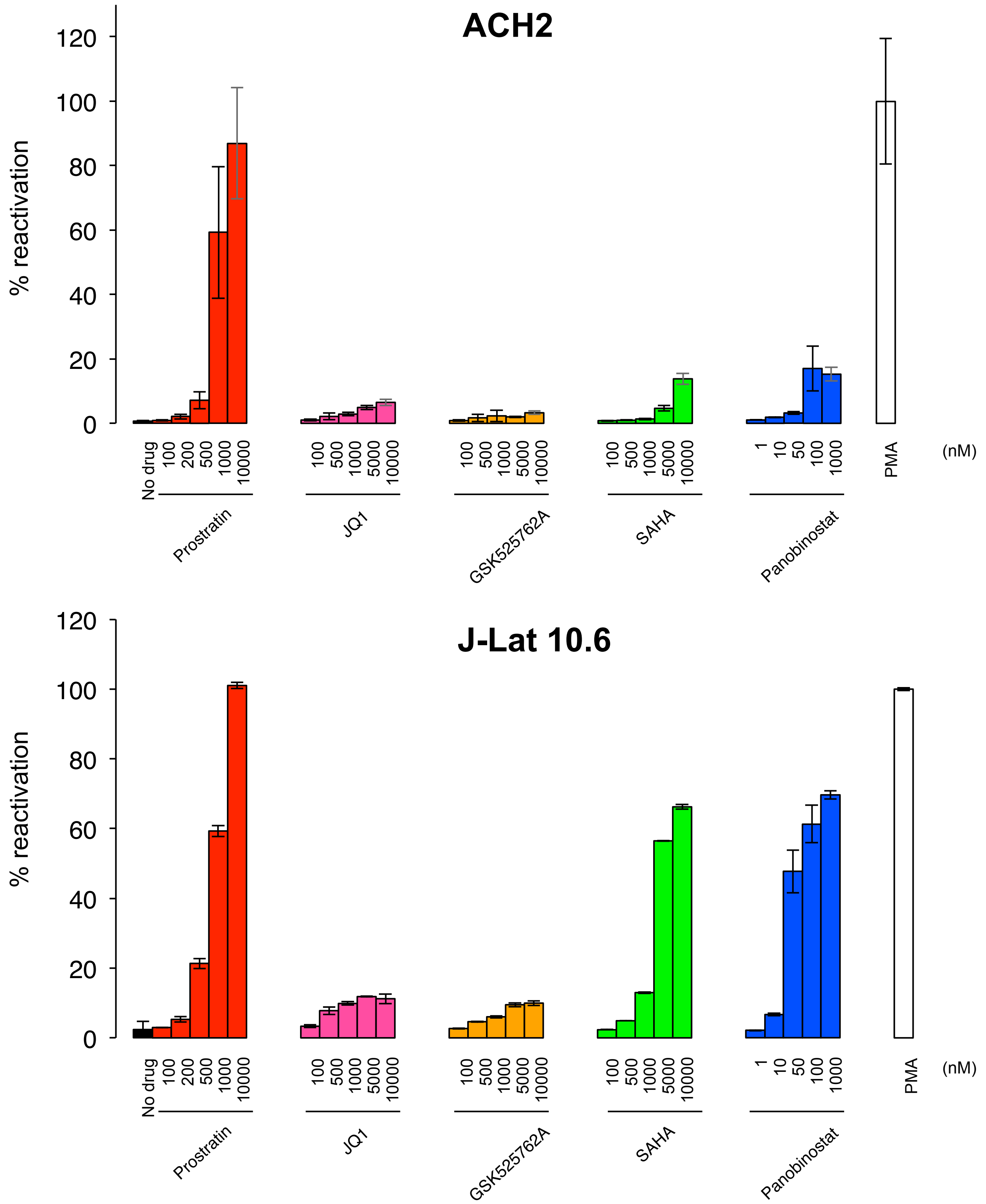

Figure S1. *In vitro* reversal of HIV-latent cells with other LRAs. HIV reversal by LRAs (prostratin, JQ1, GSK525762A, SAHA, and panobinostat) in ACH2 cells and J-Lat 10.6 was examined using same methods as experiments in Fig. 2 (*A* & *B*).
